# Emergence of Rapid-Onset Ototoxicity Following Three Doses of Vancomycin: A Unique Case Report in the Context of Normal Renal Function and Therapeutic Dosing

**DOI:** 10.7759/cureus.48647

**Published:** 2023-11-11

**Authors:** Yisroel Y Grabie, Stephanie Chain, Michael Xerras, Kelly Hong, Sadaf A Khan

**Affiliations:** 1 Internal Medicine, Staten Island University Hospital, Staten Island, USA; 2 Internal Medicine, City University of New York School of Medicine, New York, USA; 3 Pharmacology, Staten Island University Hospital, Staten Island, USA

**Keywords:** mixed hearing loss, sepsis treatment, severe sepsis, sensorinerual hearing loss, oral vancomycin

## Abstract

Vancomycin, a potent glycopeptide antibiotic renowned for its efficacy against methicillin-resistant Staphylococcus aureus, also harbors the potential for adverse reactions. While its use is often associated with infusion-related events and nephrotoxicity, ototoxicity has emerged as a noteworthy but rare concern. This adverse effect, characterized by a spectrum of transient to permanent hearing loss or damage, typically surfaces in patients receiving excessive doses, those undergoing concomitant therapy with other ototoxic agents such as aminoglycosides, or individuals with baseline hearing impairment or renal dysfunction. This report highlights the possibility of ototoxicity in the setting of normal renal function and therapeutic dosing. We report a case of a 58-year-old male patient with a complex medical history, who presented with sepsis, respiratory failure, and a constellation of underlying conditions. His treatment regimen encompassed intravenous vancomycin administration, which led to an unexpected development-severe-to-profound bilateral conductive and sensorineural hearing loss after three doses. The absence of concurrent ototoxic agents and Bayesian dosing software predicting an acceptable AUC/MIC ratio complicates the understanding of this adverse event. Amid this complex scenario, the case underscores the evolving landscape of vancomycin-induced ototoxicity, encouraging heightened vigilance, thorough audiometric monitoring, and an in-depth exploration of potential mechanisms underlying this adverse reaction. Early audiometric testing and referral to otolaryngology may allow for early intervention with high-dose steroids to mitigate the ototoxicity.

## Introduction

Vancomycin is a glycopeptide antibiotic with potent anti-staphylococcal activity commonly used for both methicillin-resistant Staphylococcal aureus and empiric coverage. Although it is most often associated with adverse drug reactions, such as infusion-related events and nephrotoxicity, warranting extended infusion durations and continuous lab monitoring, it is also associated with ototoxicity [1].

Ototoxicity can range from transient to permanent hearing loss or damage and is most commonly reported in patients with excessive doses, concomitant therapy alongside other ototoxic agents such as aminoglycosides, or baseline hearing loss or kidney dysfunction.

Vancomycin works primarily as a time-dependent antibiotic, meaning that its efficacy depends on the duration of time during which serum drug concentrations exceed the minimal inhibitory concentration (MIC). Increased concentrations past a therapeutic threshold do not result in increased bacterial killing [2].

In regard to pharmacokinetics, vancomycin has significant renal elimination with over 80-90% of the unchanged drug found in the urine over a 24-hour duration after a single dose [2]. Therefore, it requires renal dose adjustments with special monitoring considerations in those with renal impairment due to increased risk of toxicity [1].

Traditionally, peak and trough serum concentrations are used to monitor the efficacy and potential toxicity of vancomycin. However, recent studies have shown that the overall area under the curve (AUC)/MIC demonstrates a better correlation with successful outcomes with vancomycin, particularly within the recommended range of 400 and 600 mg·h/L as per the Infectious Diseases Society of America (IDSA) [3]. Although the AUC can require considerable calculations and time, Bayesian dosing software programs have been proven to generate accurate AUC estimates using at least one trough concentration. The software utilizes population pharmacokinetics with patients’ observed levels to help calculate optimal, individualized dosing regimens [3].

## Case presentation

A 58-year-old male patient with a past medical history of multiple sclerosis, treated with natalizumab infusions, complicated by a neurogenic bladder that led to multiple previous urinary tract infections with Pseudomonas aeruginosa, central diabetes insipidus, and COPD on 2.5 LPM home oxygen, presented to our hospital for the evaluation of fever and chronic bedsore on his thoracic lumbar spine. In the emergency department (ED), he met the criteria for sepsis: presenting with fever and tachycardia (Table 1). His blood pressure was normal; his SpO2 was also normal on 2 LPM oxygen through a nasal cannula. Labs demonstrated a normal serum glucose level, leukocytosis, a decreased hemoglobin level, a decreased sodium level, and a normal lactate level. A venous blood gas test was performed upon ED arrival, showing a decreased pH, increased pCO2, and a normal bicarbonate level. However, the patient became hypotensive. Urine and blood cultures were obtained, and he was empirically treated with a one-time dose of cefepime 1 g IV, a one-time dose of metronidazole 500 mg IV, and a loading dose of vancomycin 1 g IV. A home dose of desmopressin 0.1 mg twice daily was resumed. He was started on noninvasive mechanical ventilation for hypoxia and increased work of breathing. The patient was then admitted to the critical care service for acute hypoxic respiratory failure and septic shock, likely secondary to a complicated ascending urinary tract infection given the patient’s history of self-intermittent catheterization and multiple prior urinary tract infections with a positive urinalysis during this admission.

**Table 1:** Patient's Vital Signs and Laboratory Values on Admission Vital signs and laboratory values of the patient on admission. ^1^Systolic and diastolic blood pressures of the patient during the ED admission were included to reflect the change in blood pressure that led to the patient's sepsis diagnosis and subsequent transfer to the critical care unit. Laboratory values for this time were not available as they were not repeated until after the patient's unit transfer.

Variable	On Admission	During ED Admission	Reference Ranges
Temperature	101.1°F	N/A	97.7-99.5°F
Heart Rate	130 bpm	N/A	60-100 bpm
Systolic Blood Pressure	120 mmHg	90 mmHg	80-120 mmHg
Diastolic Blood Pressure	59 mmHg	40 mmHg	60-80 mmHg
Oxygen Saturation (SpO2)	95%	N/A	95-100%
White Blood Cell (WBC) Count, x 109	40.35	N/A	4.5-11
Hemoglobin	10.6 g/dL	N/A	13.8-17.2 g/dL
Glucose	75 mg/dL	N/A	70-100 mg/dL
Na+	133 mmol/L	N/A	135-145 mmol/L
Lactate	0.7 mmol/L	N/A	0.5-2.2 mmol/L
Venous Blood Gas			
pH	7.24	N/A	7.35-7.45
PCO2	64 mmHg	N/	35-45 mmHg
HCO3-	27 mmol/L	N/A	23-30 mmol/L

In the critical care unit, the patient was evaluated by the infectious disease team and the burn team who determined that the patient’s chronic pressure injury was likely not the source of his septicemia but would require a bedside debridement. The patient was then started on a standing dose of meropenem 1 g IV given every eight hours. After approximately 24 hours, he was administered vancomycin 1 g IV every 12 hours, for which he only received an additional two doses of vancomycin as the patient’s vitals stabilized, and he was downgraded to the medical floor. As advised by the infectious disease team, vancomycin was discontinued after his blood cultures returned negative, including for MRSA, and the patient was switched from meropenem to a 14-day course of cefepime 1 g IV given every eight hours. His urine culture returned positive for Pseudomonas aeruginosa, and he was continued on cefepime IV. However, he was unable to be transitioned to oral antibiotics as the susceptibilities from the urine culture demonstrated resistance to levofloxacin and ciprofloxacin.

**Figure 1:**
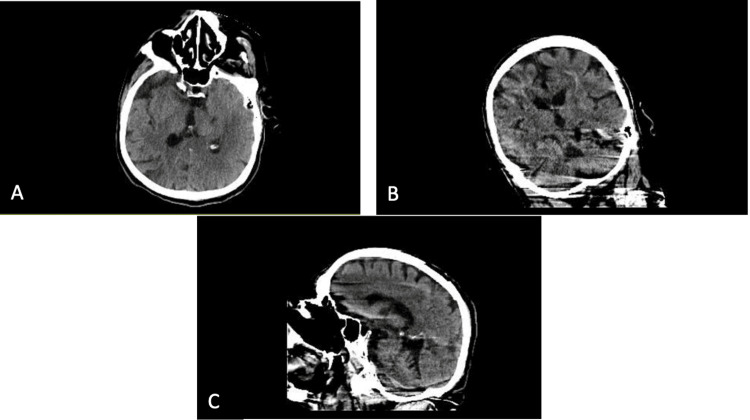
CT Scan of Head Without Contrast A. Axial View^1^ B. Coronal View^1^ C. Sagittal View^1^ 1. A CT scan of the head demonstrated prominence of the sulci, Sylvian fissures, and ventricles, likely reflecting mild diffuse parenchymal volume loss. There are scattered low attenuations in bilateral periventricular cerebral white matter consistent with mild chronic microvascular ischemic changes. There is no evidence of a large acute territorial infarct or intracranial hemorrhage. There is no large space-occupying lesion or midline shift. There is no evidence of hydrocephalus or extra-axial fluid collections. Visualized intraorbital contents are normal. Imaged portions of the paranasal sinuses and mastoid air cells are aerated. There is no evidence of a displaced skull fracture. The overall impression of the CT scan of the head was that there was no acute intracranial pathology.

On hospital day two, the patient expressed concern over his sudden hearing loss that began while he was still in the critical care unit. He denied any otalgia or tinnitus. The family was contacted and confirmed that hearing had never been a prior issue for him and that he had never required the use of amplifiers or hearing aids. Furthermore, documentation from his hospital admission two months prior was notable for a normal neurological exam without deficits. A CT scan of the head without contrast was performed during this admission, which showed no acute intracranial pathology (Figure 1). The otolaryngology team evaluated the patient and recommended manual cerumen removal and the use of cerumen removal drops to be placed in the patient’s left ear. They had also advised for an inpatient audiogram to be performed. The audiologist performed the audiogram on hospital day four, which demonstrated that the patient had severe-to-profound mixed (conductive and sensorineural) bilateral hearing loss, with the impression that the patient would have difficulty understanding clear speech in all listening conditions (Tables 2-5). The otolaryngology team then recommended a course of prednisone dosed 60 mg at minimum PO for at least five days, but it would be deferred to the medical team regarding the medication’s tapering and length. The medical team decided on the administration of prednisone 60 mg for five days without the need for tapering as the pharmacist had warned that a prolonged course of prednisone could result in hyponatremia given the synergistic effect with desmopressin needed to treat the patient’s central diabetes insipidus. As the patient continued to take prednisone, his hearing loss demonstrated much improvement; although by the end of the course, the patient still had a mild hearing deficit. The otolaryngology team advised the patient to follow up with the otolaryngologist outpatient for a repeat evaluation and audiogram. The patient was upgraded back to the critical care unit for mucus plugging, resulting in a complete white-out of his left lung, requiring mucolytics and eventually bronchoscopy. As he remained in the hospital for another month, his hearing subjectively returned to baseline.

**Table 2:** Audiogram Results: Bone Conduction Left Ear

Bone Conduction: Left Ear	
500 Hz: Unmasked	45
1000 Hz: Unmasked	15
2000 Hz: Unmasked	55
4000 Hz: Unmasked	40
500 Hz: Masked	45
1000 Hz: Masked	15
4000 Hz: Masked	40

**Table 3:** Audiogram Results: Bone Conduction Right Ear

Bone Conduction: Right Ear	
500 Hz: Masked	30
1000 Hz: Masked	40
4000 Hz: Masked	50

**Table 4:** Audiogram Results: Air Conduction Left Ear

Air Conduction: Left Ear	
250 Hz: Unmasked	90
500 Hz: Unmasked	80
1000 Hz: Unmasked	75
2000 Hz: Unmasked	70
3000 Hz: Unmasked	85
4000 Hz: Unmasked	85
6000 Hz: Unmasked	105
8000 Hz: Unmasked	NR

**Table 5:** Audiogram Results: Air Conduction Right Ear

Air Conduction: Right Ear	
250 Hz: Unmasked	70
500 Hz: Unmasked	65
1000 Hz: Unmasked	55
1500 Hz: Unmasked	65
2000 Hz: Unmasked	70
3000 Hz: Unmasked	60
4000 Hz: Unmasked	75
6000 Hz: Unmasked	85
8000 Hz: Unmasked	90

## Discussion

This case report describes a 58-year-old patient who experienced ototoxicity after three doses of vancomycin, following one loading dose of 1 g and two subsequent doses of 1 g every 12 hours. The patient had otherwise adequate renal function and a calculated creatinine clearance of 81 mL/min and was not administered any ototoxic agents, such as aminoglycosides or chemotherapeutic agents. Using Bayesian vancomycin dosing software, AUC was also predicted to be 420.4 mg·h/L, which is within the lower end of the desired therapeutic range. The extent of the patient’s ototoxicity was severe-to-profound conductive and sensorineural hearing loss as demonstrated by the inpatient audiogram.

Previous case reports of vancomycin-induced ototoxicity were published between 1958 and 1988 [4]. However, patients had either received aminoglycoside antibiotics before, during, or after vancomycin, where ototoxicity could have likely resulted from either a potentiated synergism or from the aminoglycoside alone [4]. For example, one prospective study found that among 34 patients receiving vancomycin therapy, only one of the patients developed hearing loss, and this patient was already on a gentamicin regimen [5]. Therefore, vancomycin-induced ototoxicity was disregarded by the majority of experts in 2006 [2].

However, more recent cases have been suggestive of vancomycin-induced ototoxicity by itself. A retrospective study in 2008 conducted baseline and follow-up audiometry in 89 patients receiving >14 days of vancomycin therapy. It found a significant risk of vancomycin-induced ototoxicity in those ≥53 years old (19%) versus those <53 years old (0%) (p=0.008) [6].

One study from 2015 on the therapeutic and adverse effects of vancomycin recommends that it be avoided in patients with previously diagnosed hearing loss. It suggests that patients with vancomycin-induced hearing loss may be preceded by symptoms of tinnitus prior to the demonstration of ototoxicity [7].

Finally, two cases of vancomycin-associated ototoxicity have also been reported, with the most recent case published in 2018 in a patient with normal renal function [8,9]. Although systemic absorption of oral vancomycin is often modest, this case found detectable serum vancomycin levels 24 hours after drug discontinuation. Ototoxicity was reported after the third dose and was persistent, even after reducing frequency from 125 mg every six to eight hours, which did not improve until vancomycin was stopped [8].

Overall, there is limited data regarding vancomycin-induced ototoxicity compared to that of aminoglycosides, which is better understood and established by the literature. The mechanism behind aminoglycoside-induced ototoxicity is dose-related and involves the destruction of cochlear hair cells on a cellular level, likely through the interruption of mitochondrial protein synthesis and generation of free radicals, resulting in irreversible hearing damage [10]. Vancomycin, on the other hand, is presumed to directly damage the auditory branch of the eighth cranial nerve, although the mechanism is not well understood with only a few published case reports [7].

One caveat in this case report is the patient’s diagnosis of multiple sclerosis. A case report by Tekin et al. describes a case report of sudden, reversible ototoxicity in a patient with multiple sclerosis [11]. Because our patient's condition of multiple sclerosis was managed from an outside affiliation, limited data are available to factor in or rule out the effects of the disease state regarding vancomycin-induced ototoxicity. From what was gathered from the patient's previous hospital admission documentation, the patient had been receiving natalizumab infusions for the treatment of multiple sclerosis in the outpatient setting. Although natalizumab is associated with side effects such as hypersensitivity reactions during infusion and increased risk of developing progressive multifocal leukoencephalopathy (PML), these side effects do not include ototoxicity or hearing loss [12]. Therefore, it could be safely determined that the patient's ototoxicity was most likely not a result of a delayed reaction from receiving natalizumab infusions. However, it is plausible that vancomycin and concurrent multiple sclerosis diagnosis both contributed to an increased likelihood of ototoxicity.

## Conclusions

In conclusion, this case report underscores the potential for ototoxicity associated with intravenous vancomycin, highlighting its emergence as a rare but significant concern even in cases with normal renal function and therapeutic dosing. The presented case involves a complex clinical history and an unexpected outcome of severe-to-profound bilateral mixed hearing loss after only three doses of vancomycin. This adverse event challenges conventional understanding owing to the absence of concurrent ototoxic agents and favorable Bayesian dosing predictions. As the understanding of vancomycin-induced ototoxicity evolves, heightened vigilance, thorough audiometric monitoring, and the exploration of underlying mechanisms are crucial. Early audiometric testing and timely referral to otolaryngology may enable interventions such as high-dose steroid therapy to mitigate ototoxic effects. This case calls for continued vigilance in monitoring and management strategies, particularly in cases involving critically ill patients receiving vancomycin therapy.
